# African Swine Fever Virus MGF360-14L Negatively Regulates Type I Interferon Signaling by Targeting IRF3

**DOI:** 10.3389/fcimb.2021.818969

**Published:** 2022-01-12

**Authors:** Yang Wang, Shuai Cui, Ting Xin, Xixi Wang, Hainan Yu, Shiyu Chen, Yajun Jiang, Xintao Gao, Yitong Jiang, Xiaoyu Guo, Hong Jia, Hongfei Zhu

**Affiliations:** ^1^ Institute of Animal Sciences, Chinese Academy of Agricultural Sciences, Beijing, China; ^2^ Institute of Microbiology, Chinese Academy of Sciences (CAS), Beijing, China; ^3^ Biotechnology Research Institute, Chinese Academy of Agricultural Sciences (CAAS), Beijing, China

**Keywords:** African swine fever virus, interferon, IRF3, ubiquitination, immune evasion

## Abstract

African swine fever (ASF) is a devastating infectious disease caused by African swine fever virus (ASFV). The ASFV genome encodes multiple structural and non-structural proteins that contribute to evasion of host immunity. In this study, we determined that the viral non-structural protein MGF360-14L inhibits interferon-β (IFN-β) promoter activity induced by cGAS-STING signaling. MGF360-14L was also found to downregulate expression of the IRF3 protein and promote its degradation through ubiquitin-meditated proteolysis. Moreover, MGF360-14L was shown to interact with and destabilize IRF3 by facilitating E3 ligase TRIM21-mediated K63-linked ubiquitination of IRF3. Overall, our study revealed that MGF360-14L promotes degradation of IRF3 through TRIM21, thereby inhibiting type I interferon production. These findings provide new insights into the mechanisms underlying ASFV immune evasion.

## Introduction

African swine fever (ASF) is a deadly infectious disease caused by the African swine fever virus (ASFV). ASFV is a large and complex icosahedral DNA virus that contains approximately 180∼190 kilobase pairs encoding more than 150 open reading frames (ORFs) (Alonso et al., 2018). Different strains of ASFV can cause different clinical manifestations, ranging from subclinical infection to death (Tulman et al., 2009). The main clinical signs observed in domestic pigs infected by virulent strains of ASFV include fever, hemorrhage, ataxia, and severe depression (Ge et al., 2018). As there is no commercial vaccine available, prevention and control of ASF are mainly accomplished through enhancing biosafety control or culling of infected animals (Karger et al., 2019). Current research on ASF vaccines focuses primarily on subunit vaccines or attenuated vaccines based on targeted gene deletion (Dixon et al., 2019). However, safety of the attenuated vaccine has been challenged, and further evaluation is needed.

Invading pathogens can be recognized by pattern recognition receptors (PRRs), triggering the production of type I interferon (IFN) and proinflammatory factors (Cunha, 2012). The innate immune system is equipped with many sensors to recognize viral infections, including Toll-like receptors (TLR) in cellular membranes or endosomes, Nod-like receptors (NLR) and retinoic acid-inducible gene I (RIG-I)-like receptors (RLRs) in the cytoplasm (Fitzgerald and Kagan, 2020; Lupfer et al., 2020; Onomoto et al., 2021). Cyclic guanosine monophosphate-adenosine monophosphate (cGAMP) synthase (cGAS) is currently considered the principal sensor of cytosolic DNA (Cai et al., 2014). When cGAS binds cytosolic DNA fragments, cGAS produces cGAMP through utilization of ATP and GTP, which then activates downstream stimulator of interferon gene (STING). STING recruits TANK-binding kinase 1 (TBK1) and traffics from the endoplasmic reticulum to a perinuclear endosomal compartment, leading to the activation of IFN regulatory factor 3 (IRF3) and resulting in IFN-β production (Ishikawa and Barber, 2011).

ASFV has been reported to inhibit signaling in the cGAS-STING pathway and downregulate IFN-β levels when porcine macrophages are infected with the ASFV Armenia/07 virulent strain (García-Belmonte et al., 2019). MGF-505-7R and MGF-505-11R interact with STING, inhibit the cGAS-STING signaling pathway and subvert type I IFN production (Li et al., 2021a; Yang K. et al., 2021). DP96R of the ASFV China 2018/1 strain is reported to negatively regulate type I IFN expression and NF-κB signaling by inhibiting both TBK1 and IKKβ (Wang et al., 2018). A528R inhibits TLR8-NF-κB signaling by targeting p65 activation and nuclear translocation (Liu X. et al., 2021). F317L interacts with IκB kinase β (IKKβ) and impairs NF-κB pathway activation by disrupting NF-κB activity to evade the host immune response (Yang J. et al., 2021). ASFV MGF360-14L has been selected as target gene of deletion for attenuated ASF vaccine development, yet its function is still unclear.

In this study, we determined that MGF360-14L can inhibit the production of type I IFN induced though the cGAS-STING signaling pathway. Our results show that MGF360-14L can interact with IRF3 and destabilize IRF3 by facilitating E3 ligase TRIM21-mediated ubiquitination of IRF3.

## Materials and Methods

### Cells and Virus

HEK293T and PK-15 cells were obtained from Type Culture Collection of the Chinese Academy of Science and cultured in Dulbecco’s modified Eagle medium (DMEM) containing 10% (v/v) fetal bovine serum (Gibco) and 1% penicillin-streptomycin under 5% CO_2_ at 37°C. SeV is stored in our laboratory.

### Plasmids

The full-length MGF360-14L gene of ASFV was synthesized by referring to ASFV-SY18 strain (GenBank: MH766894) and subcloned into the p3×Flag-CMV-7.1 and pCMV-N-eGFP vectors. The full-length TRIM21 gene was amplified by PCR from PK-15 cells cDNAs and cloned into vectors pcDNA3.1-HA and pcDNA3.1-Myc. The full-length IRF3 gene was amplified by PCR from PK-15 cells cDNAs and cloned into the p3×Flag-CMV-7.1 vector. IFN-β and pRL-TK luciferase reporter plasmids were purchased from Genomedi Tech (China). The hemagglutinin (HA)-tagged cGAS, STING, TBK1, and IRF3 protein expression plasmids were constructed according as previously described (Wang et al., 2018). The pGPU6-shTRIM21-852 (5’-GCTATGTGCCCGGGATTAAGA-3’) plasmid was purchased from Genepharma (China).

### Antibodies and Reagents

Rabbit monoclonal anti-IRF3, GAPDH, eGFP-Tag, HA-Tag and HRP-conjugated goat anti-rabbit IgG antibodies, and mouse monoclonal anti-Myc-Tag Flag-Tag antibody were purchased from Cell Signaling Technology (USA). The JetPRIME kit was purchased from Polyplus Transfection (France) and Double-Luciferase Reporter Assay Kit was purchased from TransGen (China). HRP-conjugated goat anti-mouse-IgG antibody, protease inhibitor, and phosphatase inhibitor were provided from CWBIO (China). The Pierce Crosslink Magnetic IP/Co-IP Kit was purchased from Thermo Scientific (USA). Goat Anti-Mouse IgG H&L (Alexa Fluor^®^ 488) and Goat Anti-Rabbit IgG H&L (Alexa Fluor^®^ 594) were purchased from Abcam (USA). TRIM21 Polyclonal antibody was purchased from Proteintech (USA). MG132, chloroquine diphosphate (CQ), 3-methyladenine (3-MA) and Z-VAD-FMK were purchased from MedChemExpress (MCE) (USA).

### qRT-PCR Assay

Total RNA extraction and cDNA synthesis were performed according to the instructions of the TaKaRa MiniBEST Universal RNA Extraction Kit and PrimeScript™ RT Master Mix (Takara). Real-time PCR was performed using a SYBR Green Master Mix (TOYOBO) in an ABI 7900HT real-time PCR system. The PCR program was as follows: 95°C for 1 min, followed by 40 amplification cycles of 95°C for 15 s, 60°C for 15 s, and 72°C for 45 s. The results are representative of three independent experiments, each performed in triplicate. The qPCR primer sequence information is as follows: Pig-GAPDH-F: 5′-CGTCCCTGAGACACGATGGT-3′, Pig-GAPDH-R: 5′-GGAACATGTAGACCATGTAG-3′; Pig-IFN-β-F: 5′-GTGGAACTTGATGGGCAGAT-3′, Pig-IFN-β-R: 5′-TTCCTCCTCCATGATTTCCTC-3′; Human-GAPDH-F: 5′- CATGAGAAGTATGACAACAGCCT-3′, Human -GAPDH-R: 5′-AGTCCTTCCACGATACCAAAGT-3′; Human -IFN-β-F: 5′-ATGACCAACAAGTGTCTCCTCC-3′, Human-IFN-β-R: 5′-GCTCATGGAAAGAGCTGTAGTG-3′ (Fan et al., 2016; Liu et al., 2018; García-Belmonte et al., 2019).

### Dual-Luciferase Reporter Assays

HEK293T cells were passed and cultured overnight on 48-well plates to 80% confluence. IFN-β-luc and pRL-TK were co-transfected with cGAS, STNG, TBK1, IRF3-5D, MGF360-14L, TRIM21, or an empty plasmid. The cell samples were collected after 24 h to detect luciferase activity using a Double-Luciferase Reporter Assay Kit according to the manufacturer’s protocol.

### IP/Co−IP and MS Assays

IP/Co-IP and MS assays were performed as follows. Briefly, HEK293T cells were transfected with the p3×Flag-MGF360-14L and pcDNA3.1-HA-TRIM21 plasmids using a JetPRIME kit (France). At 24 h after transfection, the cells were washed with ice-cold PBS. Then, ice-cold IP buffer was added to the cells and incubated on ice for 5 min with periodic mixing. The cell lysate was collected and centrifuged at 12,000×g for 10 min. The IP/co-IP was performed using a Pierce Crosslink Magnetic IP/Co-IP Kit (USA) according to the manufacturer’s protocol. Then IP/co-IP samples were analyzed by western blotting with the indicated antibodies. The p3×Flag-MGF360-14L plasmid was transfected into PK-15 cells, and the IP assay was performed. The IP protein samples were subjected to SDS-polyacrylamide gel electrophoresis, stained with Coomassie blue, and detected by MS (Novogene Bioinformatics Technology Co., Ltd., Beijing, China).

### Confocal Microscopy

PK-15 cells were transfected with the p3×Flag-MGF360-14L and pcDNA3.1-HA-TRIM21 plasmids for 24 h, fixed with 4% paraformaldehyde for 20 min at room temperature, and then permeabilized with 0.1% Triton X-100 for 15 min. The cells were incubated with anti-Flag and anti-HA tag antibody for 4°C overnight and then with Goat Anti-Mouse IgG H&L (Alexa Fluor^®^ 488) and Goat Anti-Rabbit IgG H&L (Alexa Fluor^®^ 594). The cells were observed by laser confocal microscopy after DAPI nuclear staining.

### Western Blot Analysis

HEK293T cells were lysed with radioimmunoprecipitation assay (RIPA) buffer (CWBIO), including protease and phosphatase inhibitors (CWBIO), for 10 min on ice and then centrifuged at 13,000 ×g for 10 min at 4°C. The lysed supernatant was collected and quantified using a Pierce™ BCA Protein Assay Kit (Thermo Scientific), and denatured for 10 min in 5× SDS-PAGE loading buffer (CWBIO). Proteins were separated by sodium dodecyl sulfate polyacrylamide gel electrophoresis (SDS-PAGE) and transferred to Immobilon-NC membranes (Millipore). The membranes were blocked with 5% skimmed milk or 5% bovine serum albumin (BSA) for 1 h at 37°C. Chemiluminescence detection was performed using ECL Western Blotting Substrate (Tanon).

### Statistical Analysis

All experiments were performed independently at least three times. A statistical analysis was performed using Student’s t-test (*, P < 0.05; **, P < 0.01; ***, P < 0.001; ns indicates no significance).

## Results

### MGF360-14L Inhibits cGAS/STING-Mediated IFN-β Activation

**Figure 1:**
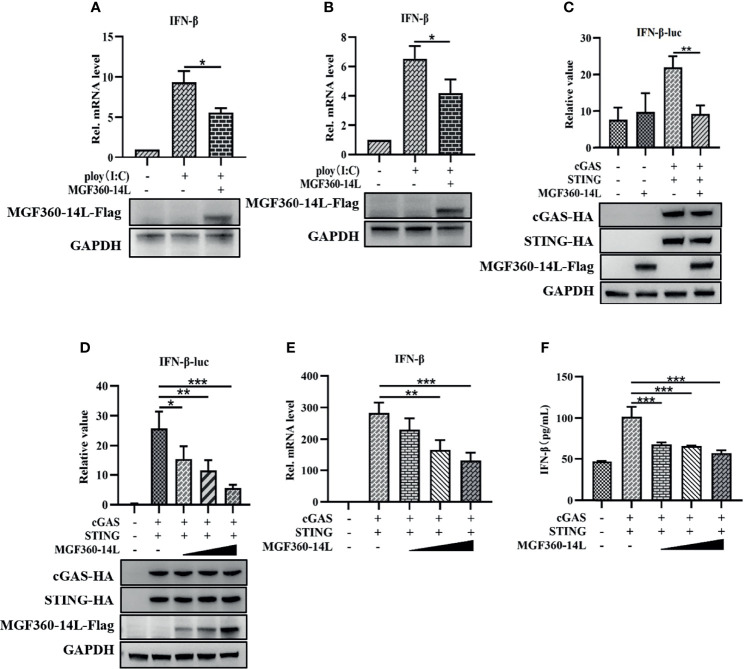
MGF360-14L inhibited IFN-β mRNA production and IFN-β promoter activity. PK-15 **(A)** and HEK293T **(B)** cells were transfected with MGF360-14L plasmids and 5 μg/mL of poly(I:C) for 24 h and then harvested for qRT-PCR assay to determine IFN-β mRNA levels. MGF360-14L inhibited cGAS/STING-mediated activation of IFN-β **(C)**, and the inhibitory effect was dose-dependent **(D)**. HEK293T cells were co-transfected with IFN-β-luc (20 ng), pRL-TK (2 ng), cGAS-HA (10 ng), STING-HA (40 ng), or the pcDNA3.1-HA empty vector (50 ng) and MGF360-14L-Flag (0 ng, 50 ng, 100 ng, 200 ng). Luciferase activity was detected at 24 h post-transfection. Expression of cGAS-HA, STING-HA, and MGF360-14L-Flag was analyzed by western blotting. **(E, F)** MGF360-14L inhibited cGAS/STING-mediated mRNA and protein levels of IFN-β. HEK293T cells were co-transfected with cGAS-HA (100 ng), STING-HA (100 ng), or the pcDNA3.1-HA empty vector (100 ng) and MGF360-14L-Flag (0 ng, 50 ng, 100 ng, 200 ng). The mRNA level of interferon-β in cell lysate was detected by qPCR, and the protein expression level of interferon-β in supernatant was detected by ELISA. Data shown are the mean ± SEM; *p < 0.001, **p < 0.001, ***p < 0.001. Representative results are from at least three independent experiments.

Previous research suggested that the virulent ASFV Armenia/07 strain reduces IFN-β mRNA expression and secretion in porcine alveolar macrophages (PAM) (García-Belmonte et al., 2019). To explore whether MGF360-14L can affect the production of IFN-β, PK-15 and HEK293T cells were transfected with MGF360-14L and treated with 5 μg/mL poly(I:C). Then, the cells were harvested and subjected to qRT-PCR to detect IFN-β mRNA. As shown in **Figures 1A, B**, MGF360-14L significantly inhibited poly(I:C)-induced IFN-β mRNA expression in PK-15 and HEK293T cells. Further, to determine whether MGF360-14L can inhibit the activity of type I IFN, MGF360-14L was co-transfected with cGAS-HA, STING-HA, and the IFN-β-luc promoter and subjected to dual-luciferase reporter assays to detect IFN-β promoter activity in HEK293T cells. The results showed that overexpression of MGF360-14L could significantly inhibit activation of the IFN-β promoter through cGAS/STING (**Figure 1C**) in a dose-dependent manner (**Figure 1D**). In addition, the results of qPCR (**Figure 1E**) and ELISA(**Figure 1F**) were consistent with the results of dual luciferase assay, which fully demonstrated the inhibitory effect of MGF360-14L on IFN-β.

### MGF360-14L Inhibits Expression of IRF3

**Figure 2:**
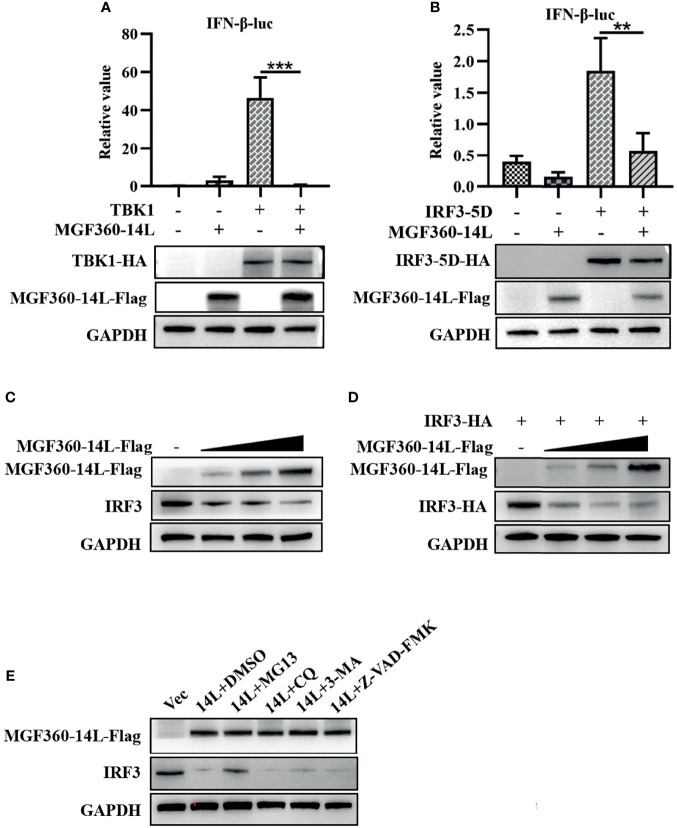
MGF360-14L inhibits IRF3-mediated IFN-β promoter activation. **(A, B)** HEK293T cells were co-transfected with IFN-β-luc (20 ng), pRL-TK (2 ng), TBK1 (50 ng) or IRF3-5D (50 ng), and MGF360-14L-Flag (50 ng) or empty plasmid. At 24 h post-transfection, cells were treated and analyzed using dual-luciferase reporter assays. **(C)** HEK293T cells were transfected with MGF360-14L-Flag (0 ng, 200 ng, 400 ng and 600 ng) or empty vector. **(D)** HEK293T cells were transfected with IRF3-HA (200 ng) and MGF360-14L-Flag (0 ng, 200 ng, 400 ng, and 600 ng) or empty vector. **(E)** HEK293T cells transfected with MGF360-14L-eGFP and then treated with DMSO or MG132 (25 μM), CQ (50 μM), 3-MA (10 mM) and Z-VAD-FMK (20 μM) for 6 h. Expression of IRF3, IRF3-HA, TBK1-HA, IRF3-5D-HA, MGF360-14L-Flag and GAPDH were assessed by western blot analysis. Data shown are the mean ± SEM; **p < 0.001, ***p < 0.001. Representative results are from at least three independent experiments.

To further explore the mechanism of MGF360-14L inhibition of IFN-β expression, MGF360-14L was co-transfected into HEK293T cells with TBK1 or the IRF3-activated mutant IRF3-5D. Then, a dual-luciferase assay was used to determine the effect of MGF360-14L on the IFN-β promoter. The results showed that MGF360-14L significantly inhibited IFN-β promoter activity stimulated by TBK1 overexpression (**Figure 2A**) and IRF3-activated mutant IRF3-5D overexpression, and MGF360-14L inhibited IRF3-5D protein expression (**Figure 2B**). These results indicated that MGF360-14L suppresses the production of IFN-β by targeting IRF3 or its downstream signals. When HEK293T cells were co-transfected with different doses of MGF360-14L and IRF3, results revealed that MGF360-14L could downregulate the expression of IRF3 (**Figures 2C, D**). Moreover, the proteasome inhibitor MG132, the lysosome inhibitor CQ and 3-MA, and the general caspase inhibitor Z-VAD-FMK were used to evaluate MGF360-14L-induced reduction of IRF3. As shown in **Figure 2E**, the proteasome inhibitor MG132 significantly restored IRF3 levels in MGF360-14L-overexpressed cells, while CQ, 3-MA and Z-VAD-FMK had no such effect on IRF3 restoration. These results indicate that MGF360-14L induces IRF3 degradation through proteasome pathway.

### MGF360-14L Interacts With TRIM21

**Figure 3:**
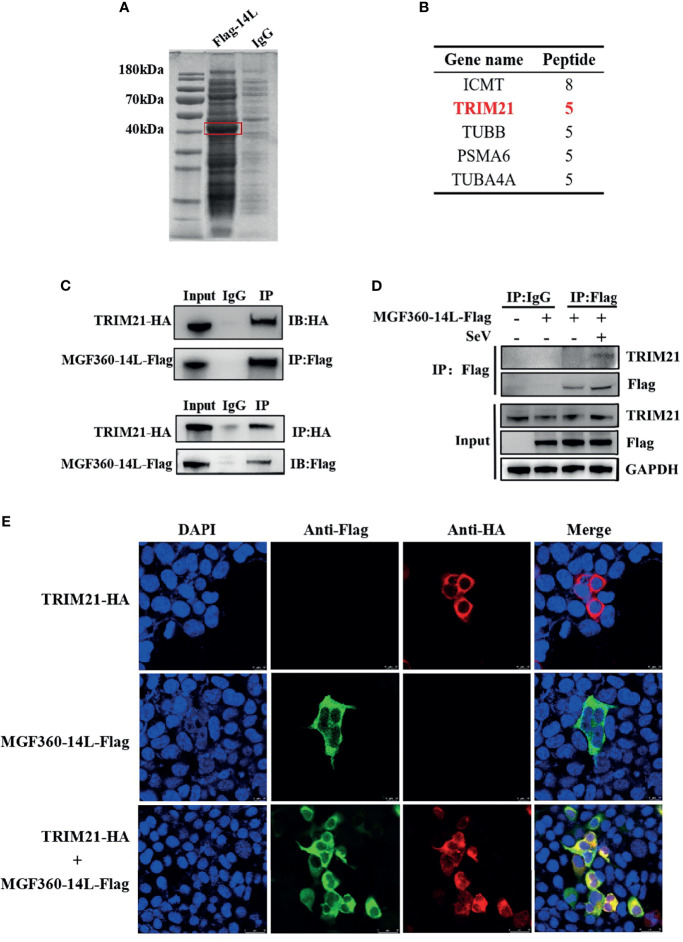
MGF360-14L interacts with TRIM21. **(A)** IP samples were separated by SDS-PAGE and stained with Coomassie blue; exogenously expressed MGF360-14L is indicated by the red box. **(B)** MS analysis of MGF360-14L-associated proteins, following by Gene Ontology (GO) analysis. High-scoring peptides were listed in the table. **(C)** HEK293T cells were transfected with MGF360-14L-Flag and TRIM21-HA plasmids. **(D)** HEK293T cells were transfected with MGF360-14L-Flag plasmids for 18 h and then incubated with SeV (1MOI) for 6 h. The cells were lysed and subjected to IP using anti-Flag, anti-HA monoclonal antibodies or control IgG. Precipitates were analyzed by western blot. **(E)** PK-15 cells were transfected with MGF360-14L-Flag and TRIM21-HA plasmids. At 24 h after transfection, cells were fixed with 4% paraformaldehyde, permeabilized with 0.1% Triton™ X-100, blocked with 5% BSA, and then treated with primary antibody and secondary antibody. Finally, laser confocal microscopy was performed after DAPI nuclear staining.

To further investigate protein targets of MGF360-14L in the IFN pathway, a CoIP-MS analysis was performed (**Figure 3A**). For the obtained proteins, we conducted a bioinformatics analysis to identify the top five interaction proteins and determine the confidence values associated with each (**Figure 3B**). The tripartite motif protein 21 (TRIM21) plays a regulatory role in innate immune responses by interacting with antiviral factors. Studies have shown that TRIM21 has a wide range of regulatory effects on IRF3-mediated type I IFN (Stacey et al., 2012; Tan and Xia, 2020). To verify interaction between MGF360-14L and TRIM21, HEK293T cells and PK15 were co-transfected with MGF360-14L-Flag and TRIM21-HA plasmids, respectively. At 24 h post co-transfection, cell samples were collected and fixed for later Co-IP assay and confocal imaging. Co-IP analysis confirmed the interaction between MGF360-14L and TRIM21 (**Figure 3C**). MGF360-14L also interacted with endogenous TRIM21 in SeV-infected HEK293T cells (**Figure 3D**). Laser confocal results showed had extensive intracellular co-localization of MGF360-14L with TRIM21 in the cytoplasm (**Figure 3E**). These results indicate that MGF360-14L can interact with endogenous and exogenous TRIM21 and co-localize in the cytoplasm.

### MGF360-14L Destabilizes IRF3 by Facilitating TRIM21-Mediated Ubiquitination of IRF3

**Figure 4:**
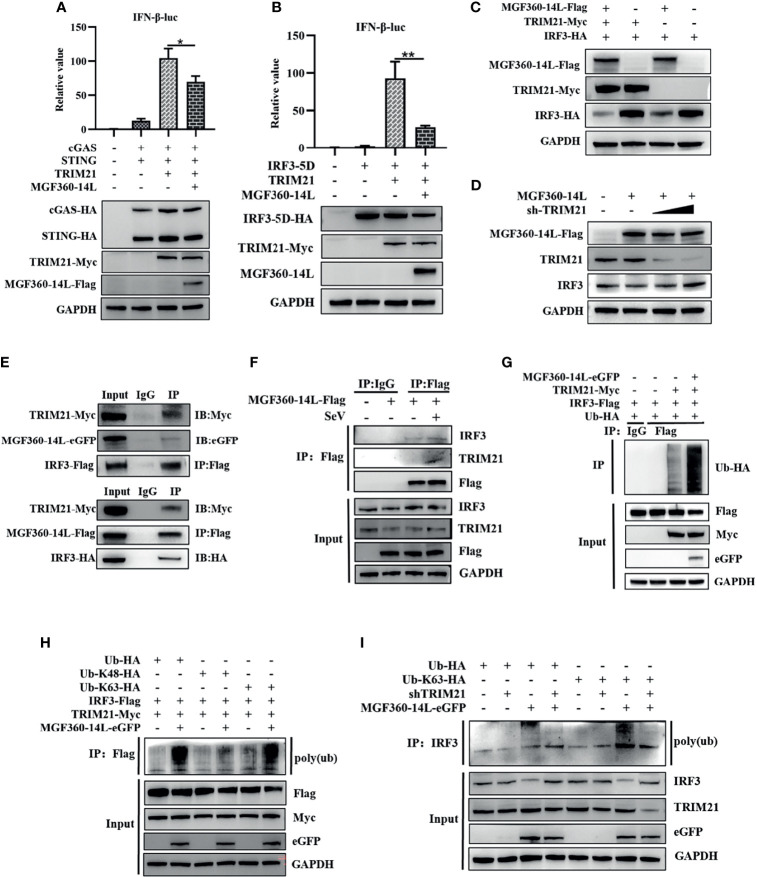
MGF360-14L destabilizes IRF3. **(A, B)** HEK293T cells were co-transfected with IFN-β-luc (20 ng), pRL-TK (2 ng), cGAS (10 ng), STING (40 ng) or IRF3-5D-HA (50 ng), MGF360-14L-Flag (50 ng), and TRIM21-HA (50 ng) or empty plasmid. At 24 h post-transfection, cells were treated and analyzed using dual-luciferase reporter assays. **(C)** HEK293T cells were transfected with MGF360-14L-Flag (500 ng), TRIM21-Myc (500 ng), IRF3-HA (500 ng) or empty plasmids. **(D)** HEK293Tcells were transfected with MGF360-14L-Flag (500 ng), sh-TRIM21 (500 ng,1000 ng) or empty plasmid. **(E)** HEK293T cells were transfected with MGF360-14L-eGFP (or MGF360-14L-Flag), TRIM21-Myc and IRF3-Flag (or IRF3-HA) plasmids. **(F)** HEK293T cells were transfected with MGF360-14L-Flag plasmids for 18h and then incubated with SeV (1MOI) for 6 h. The cells were lysed, and subjected to IP using anti-Flag or control IgG. Precipitates were analyzed by WB. **(G, H)** IP with anti-Flag beads analysis of lysates of HEK293T cells transfected with Ub-HA, or Ub-K48-HA, or Ub-K63-HA, TRIM21-Myc, MGF360-14L-eGFP and IRF3-Flag for 24h. **(I)** IP with anti-IRF3 beads analysis of lysates of HEK293T cells transfected with Ub-HA, or Ub-K63-HA, shTRIM21 and MGF360-14L-eGFP for 24h. Expression of MGF360-14L-Flag (or MGF360-14L-eGFP), TRM21-Myc (or TRIM21-HA), IRF3-HA (or IRF3-Flag),Ub-HA, Ub-K48-HA, Ub-K63-HA and GAPDH were assessed by Western blot analysis. Data shown are the mean ± SEM; *p < 0.001, **p < 0.001. Representative results are from at least three independent experiments.

TRIM21 can interact with ASFV MGF360-14L. However, whether TRIM21 affects MGF360-14L-mediated inhibition of IFN-β remains unknown. To investigate the inhibitory effects of TRIM21, TRIM21 was co-transfected with cGAS-HA, STING-HA, IRF3-5D-HA, IFN-β-luc promoter, and MGF360-14L-Flag into HEK293T cells, and then subjected to dual-luciferase reporter assay to detect IFN-β promoter activity. The results showed that although TRIM21 could significantly promote IFN-β promoter activation through cGAS/STING and IRF3-5D, MGF360-14L still had an inhibitory effect (**Figures 4A, B**). When TRIM21-Myc, IRF3-HA, and MGF360-14L-Flag were co-transfected into HEK293T cells, IRF3 protein levels were considerably decreased compared to that following TRIM21 and IRF3-HA co-transfection (**Figure 4C**). More importantly, the inhibition of IRF3 by MGF360-14L was weakened by silencing endogenous TRIM21(**Figure 4D**). In addition, Co-IP and western blot assays showed that MGF360-14L interacted with endogenous and exogenous TRIM21 and IRF3 (**Figures 4E, F**). A previous study showed that TRIM21 can promote ubiquitination of IRF3, thus inhibiting the production of type I IFN (Ma et al., 2021), and our research showed that ubiquitination of IRF3 was significantly increased when MGF360-14L and TRIM21 were co-transfected in HEK293T cells compared with that following transfection with TRIM21 alone (**Figure 4G**). Additionally, MGF360-14L enhanced TRIM21-mediated K63-linked but not K48-linked ubiquitination of IRF3 (**Figure 4H**). After further silencing of TRIM21, the ubiquitination of endogenous IRF3 promoted by MGF360-14L was significantly weakened (**Figure 4I**). In conclusion, our results indicate that MGF360-14L can interact with IRF3 and destabilize IRF3 by facilitating E3 ligase TRIM21-mediated K63-linked ubiquitination.

## Discussion

Innate immune responses are the first line of defense against invading pathogens. Among cytosolic nucleic acid sensors, cGAS plays an essential role in the recognition and inhibition of DNA viruses. Some DNA viruses can evade recognition through the cGAS-STING pathway (Verrier and Langevin, 2021). For instance, the herpes simplex virus 1 (HSV-1) tegument protein VP22 interacts with cGAS and counteracts cGAS/STING-mediated DNA-sensing antiviral innate immune signaling by inhibiting cGAS enzyme activity (Huang et al., 2018). HSV-1 UL46 impairs interactions between TBK1 and IRF3 and downregulates IRF3 activation to reduce IFN-I production (You et al., 2019). Human cytomegalovirus (HCMV) IE86 protein facilitates the proteasome-dependent degradation of STING and blocks the cGAS-STING pathway (Kim et al., 2017). Virulent VACV strains inhibits STING dimerization and phosphorylation during infection, efficiently suppressing DNA sensing and IRF-3 activation (Georgana et al., 2018). ASFV is also known to target the cGAS-STING pathway and downregulate IFN-β levels (García-Belmonte et al., 2019).

IRF3 is essential for the induction of type I IFN and stimulation of innate immune responses. Viruses adopt multiple strategies to inhibit IRF3 activation to evade the host’s innate antiviral immune responses. For instance, dengue virus and foot-and-mouth disease virus can inhibit type I IFN by reducing IRF3 phosphorylation (Dalrymple et al., 2015; Yang et al., 2019). Peste des petits ruminants virus has been shown to block interactions between TBK1 and IRF3, interfering dimerization of IRF3 and nuclear transportation (Li P. et al., 2021). In addition, other viral proteins can inhibit IFN-β expression by degrading IRF3. For example, Seneca Valley virus 3C^pro^ interacts with IRF3 and reduces IRF3 protein expression, allowing the virus to escape the host innate immune responses (Xue Q. et al., 2018). The viral protease SARS-CoV-2 3CL was found to attenuate type I IFN production by inhibiting the nuclear translocation of IRF3 and promoting IRF3 degradation (Zhang et al., 2021). The pseudorabies virus kinase UL13 inhibits IFN-β signaling by targeting IRF3 for ubiquitination and degradation (Lv et al., 2020). In this study, we demonstrated MGF360-14L-mediated degradation of IRF3 through the ubiquitin proteasome pathway leading to inhibition of IFN-β expression.

Several proteins encoded by ASFV play important roles in sabotaging host defense mechanisms that are critical immune evasion by the virus (Correia et al., 2013). For instance, DP96R of ASFV China 2018/1 was reported to negatively regulate IFN-I expression and NF-κB signaling by inhibiting both TBK1 and IKKβ (Wang et al., 2018). MGF-505-7R interacts with IKKα, STING, and IRF3 and inhibits the cGAS-STING signaling pathway to block IFN-β and inhibit pro-inflammatory IFN-γ-mediated JAK-STAT1 signaling (Li J. et al., 2021; Li et al., 2021a; Li et al., 2021b). E120R interacts with IRF3 and interferes with recruitment of IRF3 to TBK1, which in turn suppresses IRF3 phosphorylation, decreasing IFN production (Liu H. et al., 2021). MGF360-12L inhibits type I IFN production by blocking interactions between importin α and proteins in the NF-κB signaling pathway (Zhuo et al., 2021). Whether or not ASFV encodes other immune escape genes remains to be determined. In this study, we revealed inhibition of the host antiviral immune response by the ASFV non-structural protein MGF360-14L through its targeting of IRF3 for degradation.

Our data indicate that MGF360-14L can significantly inhibit type I IFN signaling. However, previous studies have shown that the deletion of MGF360-13L and MGF360-14L alone in Georgia/2007 strain did not affect virus replication in cell cultures and disease progression in swine (Borca et al., 2017). ASFV is a large and complex DNA virus, encoding a variety of proteins, which perform the repetitive or overlapping functions.

In this study, MGF360-14L inhibited poly(I:C)-induced IFN-β mRNA expression (**Figures 1A, B**) and activation of the IFN-β promoter through cGAS/STING(**Figure 1C**), TBK1(**Figure 2A**) and IRF3-activated mutant IRF3-5D (**Figure 2B**). Western blot results revealed that MGF360-14L downregulates the expression of endogenous and exogenous IRF3 for significant dose-dependent inhibition (**Figures 2C, D**). Moreover, MGF360-14L promoted degradation of IRF3 through ubiquitin-meditated proteolysis (**Figure 2E**). Further analysis confirmed MGF360-14L interaction with endogenous and exogenous TRIM21 and co-localization in the cytoplasm (**Figures 3C–E**). TRIM21 is a E3 ubiquitin ligase that plays an important role in innate immune regulation (Lee, 2017). For example, TRIM21 enhances herpes simplex virus-1 replication in corneal epithelial cells by suppressing the production of type I IFN through inhibition of STING/IRF3 signaling (Stacey et al., 2012). TRIM21 promote innate immune responses to RNA virus infection through Lys27-linked polyubiquitination of MAVS (Xue B. et al., 2018). HPV E7 recruits the E3 ligase TRIM21 to ubiquitinate and degrade the IFI16 inflammasome, leading to inhibition of cell pyroptosis and escape from immune surveillance (Song et al., 2020). Similarly, our results showed that MGF360-14L interacts with IRF3 and promotes the K63-linked polyubiquitination of IRF3 by recruiting the E3 ligase TRIM21, resulting in inhibition of type I IFN (**Figures 4E–I**). Unfortunately, we did not verify the effect of MGF360-14L on IFN-β at the viral level. These findings reveal a novel strategy for ASFV immune evasion and suggest new targets for ASFV vaccine research.

## Data Availability Statement

The original contributions presented in the study are included in the article/supplementary material. Further inquiries can be directed to the corresponding authors.

## Author Contributions

YW, SCu, and XGu designed the research. YW, SCh, HYu, and YaJ performed the experiments. YW and SCu analyzed the data. YW and SCu wrote the manuscript. All authors discussed results and reviewed the manuscript. All authors contributed to the article and approved the submitted version.

## Funding

This work was supported by the National Key R&D Plan of China (2021YFD1800008) and Yunnan Key Research and Development project (202103AC100001).

## Conflict of Interest

The authors declare that the research was conducted in the absence of any commercial or financial relationships that could be construed as a potential conflict of interest.

## Publisher’s Note

All claims expressed in this article are solely those of the authors and do not necessarily represent those of their affiliated organizations, or those of the publisher, the editors and the reviewers. Any product that may be evaluated in this article, or claim that may be made by its manufacturer, is not guaranteed or endorsed by the publisher.
